# Asexual reproduction in reef-building corals: insights into fragment attachment to improve restoration and predict natural recovery

**DOI:** 10.1098/rsos.251209

**Published:** 2025-10-29

**Authors:** Brett Maxwell Lewis, David Suggett, Peter Prentis, Crystal Cooper, Luke D. Nothdurft

**Affiliations:** ^1^ Department of Earth and Atmospheric Science, Queensland University of Technology, Brisbane, Queensland, Australia; ^2^ Coral Restoration Initiative (KCRI), King Abdullah University of Science and Technology, Thuwal, Makkah, Saudi Arabia; ^3^ Climate Change Cluster (C3), University of Technology Sydney, Sydney, New South Wales, Australia; ^4^ School of Biology and Environmental Science, Queensland University of Technology, Brisbane, Queensland, Australia; ^5^ Centre for Agriculture and Bioeconomy, Queensland University of Technology, Brisbane, Queensland, Australia; ^6^ Central Analytical Research Facility, Queensland University of Technology, Brisbane, Queensland, Australia; ^7^ Centre for Microscopy Characterisation and Analysis, University of Western Australia, Crawley, Western Australia, Australia; ^8^ School of Earth and Atmospheric Sciences, Queensland University of Technology, Brisbane, Queensland, Australia

**Keywords:** coral cellular biology, trait-based coral reef restoration, electron, light and fluorescence microscopy, coral evolution and adaptation, coral microstructural development, coral fragment substrate attachment, coral ultrastructure development and behaviour, reef-building coral emergence, asexual reproduction in reef-building coral, strategic decisions on transplanting schedules, resource allocation and monitoring in reef restoration

## Abstract

Coral reefs are experiencing global decline, and their recovery relies heavily on asexual reproduction through fragmentation, the success of which hinges on self-sustaining attachment to the reef substrate. However, despite decades of research into coral biology, we still lack a comprehensive understanding of the attachment process and how to optimize efforts exploiting it. We recently proposed a model explaining the attachment process in *Acropora millepora* (Lewis BM, Suggett DS, Prentis PJ, Nothdurft LD. 2022 Cellular adaptations leading to coral fragment attachment on artificial substrates in *Acropora millepora* (Am-CAM). *Sci. Rep*. **12**, 18431. (doi:10.1038/s41598-022-23134-8)). To determine if the model is conserved across coral species, we employed cutting-edge integrated optical and electron microscopy techniques to observe attachment development in two key reef-forming coral genera, including *Montipora mollis* and *Pocillopora verrucosa*, comparing them with the previous model, *A. millepora*. Although developmental steps were broadly conserved, we identified taxonomically distinct variations in immune responses, behaviour, tissue development and skeletal microstructure. These differences explain why certain coral species, like *M. mollis* and *A. millepora*, can exhibit faster and stronger attachment compared with *P. verrucosa.* These findings provide critical diagnostics for asexual success and offer actionable insights into coral fundamental biology and for enhancing reef restoration efforts.

## 1. Introduction

Coral reef ecosystems undergo disturbance cycles that influence the loss and recovery of reef-building corals [1–4]. Accelerating pressures from climate change and local stressors continue to magnify these disturbance cycles, leading to a net decline of coral cover by 50% over the last 75 years [5]. Reef managers and stakeholders face the increasing challenge of mitigating stressors to minimize further coral loss while maximizing coral population recovery. While addressing climate change is crucial for the sustained recovery of declining reefs, restoration-based interventions that actively repopulate coral biomass [6–8] can be coupled with substrate stabilization [9,10] to provide critical restoration initiatives that aid interim recovery [11,12].

Global coral reef restoration relies heavily on planting strategies that leverage both sexual reproduction (larvae produced from annual spawning events) and asexual reproduction (attachment of broken fragments from established coral colonies to the reef substrate) [13,14]. Asexual-based methods for coral restoration generally involve two key techniques: (i) nursery rearing of coral fragments on suitable substrates (e.g. ceramic, aragonite) until they develop sufficient tissue or reach a designated transplantation period, and (ii) direct transplantation, where these fragments are attached to the reef. Asexual propagation has gained prominence globally due to its broad applicability year-round, its cost-effectiveness, low training requirements and subsequent ecotourism appeal [7,14]. Nevertheless, for any propagation mode, attachment to reef substrates operates as a fundamental bottleneck to success [15–18].

Coral transplantation relies on artificial attachment methods such as epoxy resins, cements, Coral Clip® or zip ties that create physical barriers to establish an initial attachment. In many cases, coral must grow over the attachment device to reach the reef substrate, increasing the time until transplant attachment is successful and slowing the formation of a self-sustaining coral base able to resist external hydrodynamics and physical forces that would dislodge the fragment without artificial attachment mechanisms [1]. Moreover, transplant detachment can be exacerbated by local environmental factors such as substrate conditions or high wave exposure. Presently, our current understanding of coral basal attachment biology and the fundamental biological factors that limit the rate and effectiveness of its development is limited [1].

Currently, the appearance of the first basal tissues is a commonly used qualifier to define attachment during coral fragment outplanting [19–21]. However, this definition of attachment may be insufficient due to differences between skeletal microstructure that develop at the fragment–substrate interface, which could lead to variations in attachment strength through time, and therefore resilience in the ecosystem, regardless of the amount of tissue present [1]. For example, according to Guest *et al*. at least one point of attachment had formed at approximately 16–24 days, which is faster than both *Montipora digitata* and *Pocillopora damicornis* (one attachment point after approx. 32–113 and 83−116 days, respectively) [19]. However, when observing the microscopic development of the basal development, formation of the soft tissue occurs before that of a robust skeleton [1]. As such, the fragment may not be securely attached to the surface or self-sustaining [19]. This development is a process that could vary across species, much like tissue attachment rates [19,22,23]. Directly observing the substrate–fragment interface to monitor tissue, cell ultrastructure and skeletal development is critical for understanding asexual reproduction and optimizing conditions for both fast- and slow-attaching species, enabling more accurate predictions of natural recovery and informing strategic decisions on transplanting schedules, resource allocation and monitoring.

The scarcity of ultrastructural studies across diverse coral species has homogenized our understanding of coral biology, overlooking critical taxon-specific processes. Much of our understanding of coral tissues and cell ultrastructure comes from a limited number of coral species within families like *Acroporiidae* and *Pocilloporidae*, despite there being 47 families and about 1678 species of stony corals [24]. As a result, there are significant gaps in our knowledge of basic coral biology [24–27]. Recently, for example, new methods used a suite of state-of-the-art microscopy *in vivo* to establish a Coral Attachment Model (CAM) describing coral fragment basal attachment development by observing the interface between the substrate and coral fragments of *A. millepora* [1]. Three distinct developmental phases were characterized: (Phase 1) contact response to initiate soft-tissue growth and protect the fragment from infection and disease, followed by (Phase 2) fragment stabilization through anchoring by the soft tissue, and then (Phase 3), where [19] a ‘lappet-like appendage’ formed at the basal rim to produce the skeleton and to allow encrustation. Not only did this study describe these phases in detail, but it also documented undescribed cells, appendages and biological processes in what is one of the most investigated coral species. Such new findings suggest that significant discoveries remain in coral biology, particularly across species and genera, but also our broadly homogenized view of coral ultrastructure and tissue function may vary more than previously expected. Investigating coral tissue ultrastructure could therefore offer insights into coral resilience and growth, in addition to attachment processes [19].

We used advanced multimodal optical *in vivo* live-cell and fixed fluorescence and Scanning Electron Microscopy (SEM) techniques [26,28–32] to produce high-resolution time-resolved visualizations of the coral–substrate attachment interface of two species with known differences in substrate attachment rates (*M. mollis* and *P. verrucosa*). The data was then compared with *A. millepora* to identify whether these three different coral species adhere to a conserved ‘model’ for substrate attachment and to devise more suitable classification features for successful coral attachment to improve reef restoration and our understanding of basic coral biology.

## 2. Methodology

### 2.1. Coral collection and experimental aquaria

Coral colonies of *Montipora mollis* (*n* = 4) that had a combined columnar and plating growth morphology, as well as colonies of *Pocillopora verrucosa* (*n* = 5) with thick and compact branches, were sourced from coastal waters off the Abrolhos Islands (28°43 S, 113°47 E) (*M. mollis n* = 4, *P. verrucosa n* = 5). Colonies were collected by Batavia Coral Farms (Geraldton, Australia). Colonies were housed and acclimated together in laboratory aquaria at the University of Technology Sydney (UTS). The 1524 mm × 610 mm × 610 mm system with biological sump housed artificial seawater (Instant Ocean, USA) at a salinity of 35 PSU, monitored daily with a digital refractometer (HI 96800, Hanna Instruments, Woonsocket, RI, USA), and maintained at 26 ± 0.5°C. Water flow was sustained at 1200 l per h using a Gyre XF 150 adjustable wavemaker (Maxspect, Wanchai, Hong Kong). Light was provided by LEDs (Aqua Illumination, Pennsylvania, USA) on a 10 h:14 h light:dark cycle peaking in intensity around midday (4 h) at 300 µmol m^−2^ s^−1^, measured at the coral surface using an underwater quantum flux meter (Apogee, Utah, USA). Key water chemistry parameters were maintained at target levels—calcium (450 ± 10 ppm), magnesium (1350 ± 50 ppm) and alkalinity (135 ± 10 ppm)—using automated dosing pumps. The dosing regimen was adjusted as needed based on weekly measurements with a commercial test kit (Reef Foundation Pro, Red Sea, USA).

**Figure 1.**
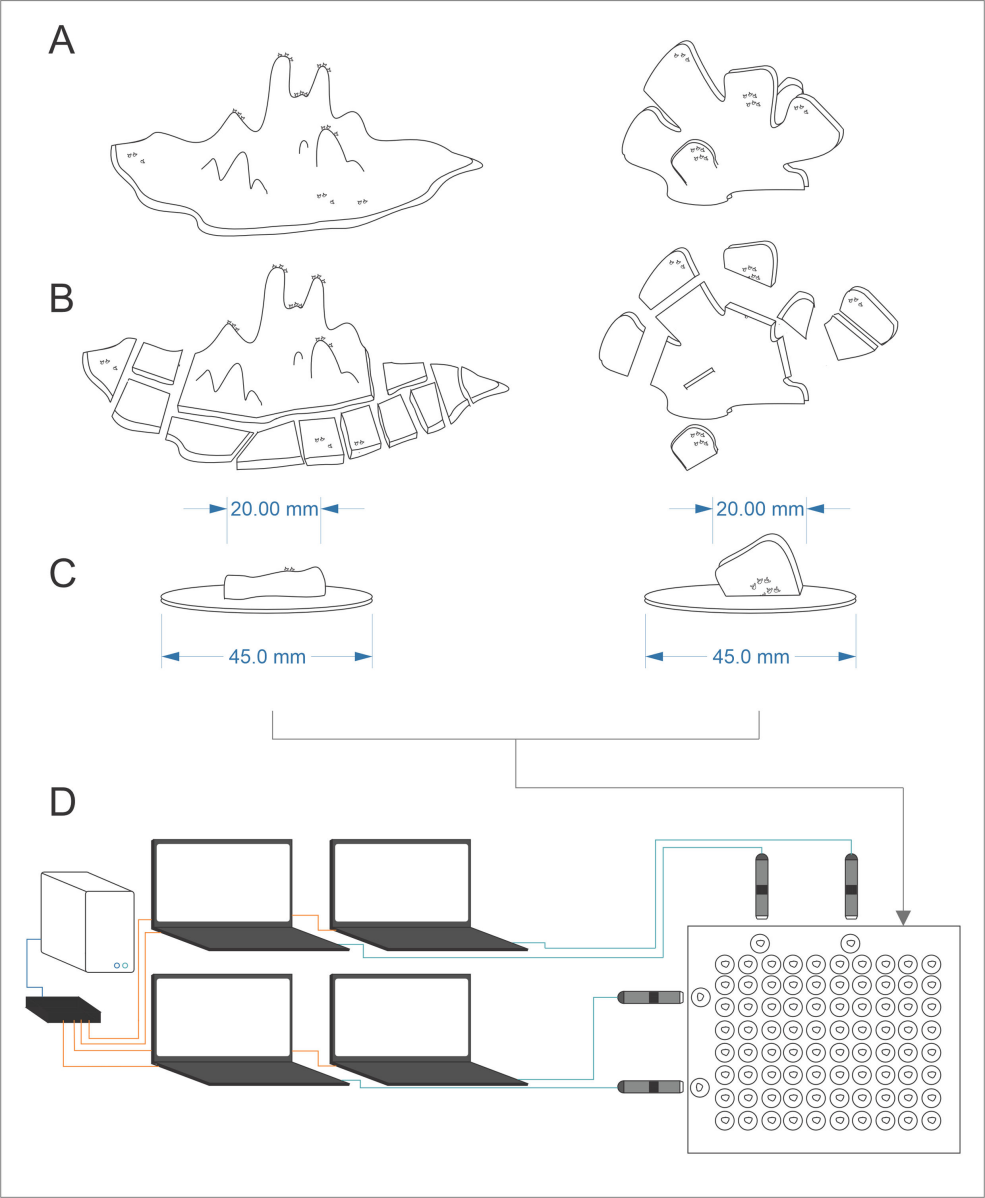
Graphic showing the fragmentation process and the experimental setup. New colonies (A) were collected and acclimated before being fragmented (B) into smaller pieces and placed onto ceramic discs (C). Tissue recovery on the fragments was tracked using macro imaging and UV light. Fragments still recovering after 10 days were not used for the trials. Attachment of the fragments to the substrate was tracked using light microscopy (D) for the duration of the trials. Each microscope was controlled by an individual computer and data (>5000 h) was collated and stored using a specifically designed portable server system (D).

Following an initial acclimation period of 10 days, each colony was divided into 30 mm to 40 mm fragments (*n* = 40 per species) using a Dremel Cordless Rotary Tool (Bosch, USA) (figure 1). For *M. mollis,* fragments were collected from plating areas of the colony due to the ease of segmentation, as these were areas undergoing growth and were most likely to grow naturally. All fragments were acclimated for a further 10 days [33] to recover from fragmentation. Green fluorescent proteins (GFP) in the recovering tissue illuminated by ultraviolet (UV) light (370 nm) were used to track the growth of new tissue. All fragments were maintained on the glass bottom of the tank and gently repositioned every 1 to 2 days to ensure that no fragments began attachment before experimentation. Fully recovered fragments were placed and observed attaching onto inert circular ceramic plates of consistent size (45 mm in diameter), shape and surface texture (figure 1). Each ceramic disc was kiln-fired at 2000°C at production to remain chemically and biologically inert and ensure a standardized substrate.

Four trials were first conducted using clear glass slides as a substrate for both species. This technique was used to directly examine the interface between the fragment and substrate colony, which was otherwise obstructed by the ceramic plate and fragment shape. Overall, the timelapse resulted in two complete and three partial datasets in *M. mollis* and one complete and four partial datasets for *P. verrucosa*. Day 1 was considered the first point of contact of coral with the ceramic substrate. The glass and ceramic substrates were positioned at the edge of the colony or the surface/base of the fragment in a way that ensured contact with established tissues and not recovered ones.

### 2.2. Time-lapse light microscopy

Time-lapse light microscopy was used to observe processes on the coral surface during the experiment, including soft tissue behaviour and gross anatomy. Time-lapse videos were captured using a Dino-Lite AM7915 portable light microscope (Dino-Lite, New Taipei City, Taiwan) positioned outside the aquaria. Samples selected for observation were positioned close to the aquarium glass wall within the microscope focal distance (figure 1). Images were captured every 30 s for 20 to 45 days, with Playback set at 15 frames per second.

### 2.3. Sample preparation

Sampling methods were adapted from previous studies for Stereo light microscopy, Confocal Laser Scanning Microscopy (CLSM) and Scanning Electron Microscope (SEM) to observe both coral tissue and skeleton. Two coral fragment samples were removed from the aquarium every 2 days over a 30 day experimental period for *M. mollis* and a 45 day experimental period for *P. verrucosa*. Samples were fixed in a solution comprising 3% paraformaldehyde, 3% glutaraldehyde and saltwater for a minimum of 30 days, with each sample size ranging from 30 mm to 45 mm. Each fixed sample was placed into 1× PBS for longer-term storage at 4°C.

Subsequently, one non-stained sample from each timepoint was cut in half. One half was dehydrated using a series of ethanol and embedded in resin for CLSM without heavy metal staining to retain natural auto-fluorescence. The dehydration involved gradually increasing ethanol concentrations in water (10%, 20%, 30%, 40%, 50%, 60%, 70%, 80%, 90%, 100%). Each dehydration step was processed in a Biowave (Pelco, USA) and repeated twice with 20 min between each step. Upon completing the ethanol dehydration step, the samples were dehydrated in 100% acetone (repeated twice), with the final treatment left overnight. Following infiltration with acetone, the samples underwent a series of 30 min resin infiltration steps with gradually increasing concentrations of Epon resin (for CSLM/SEM) in acetone, at 10%, 20%, 40%, 50%, 70%, 90% and 100%. The 100% resin step was repeated three times, with resin infiltration at 70%, 90% and 100% being left overnight. Samples were cured at 60°C for at least 48 h.

The other sample half was treated with a series of aqueous heavy metal stains following fixation to improve the quality of the SEM micrographs. The heavy metals used included 2% osmium tetroxide in 1.5% potassium ferricyanide for a minimum of 1 h, thiocarbohydrazide for 30 min, 2% osmium tetroxide for 30 min, 1% uranyl acetate overnight at 4°C and finally lead aspartate (20 mM lead nitrate in 0.03 M L-aspartic acid adjusted to pH 5.5 with 1 M NaOH) for 1 h adapting previously described methods [34–36]. The samples were washed with water for 5 min between each stain before being dehydrated and infiltrated with resin using the same protocols as unstained samples.

Once set, all resin blocks (CLSM/SEM) were cut to expose a cross-section of the coral–substrate interface (figure 1) using a diamond saw. The cross-sectioned surfaces were polished using wet/dry sandpaper (CAMI, from 400 to 2000 grit) and finished using a lapidary polishing wheel and 1 µm aluminium oxide compound on a cloth pad. Samples were then rinsed using deionized water (DI) and placed into an ultrasonic cleaner to remove fine particles from the surface. Samples were etched using 1% formic acid solution for approximately 10 s before rinsing in DI to expose the skeletal microstructure for SEM.

Following auto-fluorescence analysis in the CLSM of non-stained samples, any regions of interest underwent en-bloc staining to improve contrast for SEM. Sample surfaces were exposed to 1% uranyl acetate for 10 to 15 min. The area was then rinsed with DI water before being stained with lead citrate for an additional 10 to 15 min. These steps were repeated up to five times or until the tissues became visibly darker. For samples grown onto glass substrates, the tissues were removed and the dry skeletons were used in additional SEM.

### 2.4. CLSM

**Figure 2.**
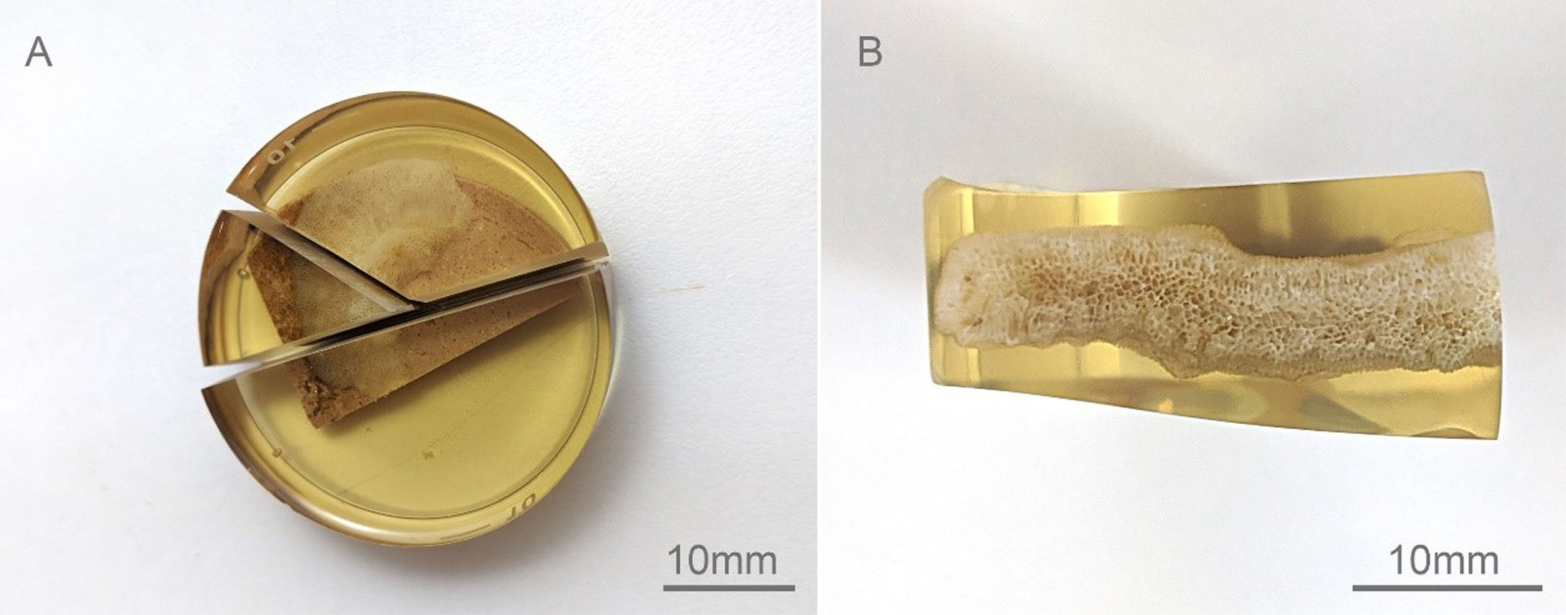
Resin embedded coral fragment on the attaching rim in *M. mollis* showing the (A) cut resin sample and (B) cross-sectioned surface to be analysed.

Microanatomy and auto-fluorescence of the coral fragments embedded in resin (figure 2) were examined using an CLSM A1R HD25 (Nikon, Tokyo, Japan) with excitation wavelengths of 405 nm (blue), 488 nm (green), 561 nm (red) and 640 nm (deep red) in a N4S 4-laser unit. Emission wavelengths range from 425–475 nm (blue), to 500–550 nm (green), 500–620 nm (red) and 663–738 nm (deep red). Laser power was set at 1−1.5 for 10× to 20× objectives and 2–4 for 40× to 60× objectives. Depending on the objective used, the gain ranged between 90−115 for blue, green and red emissions, or between 115−140 for weaker deep red emission. Images were collected at digital pixel resolutions ranging from 2540×1600–3840 ×2160. For volume imaging, each section was taken at approximately 5 nm. NIS-Elements CLSM (Nikon, NY, USA) spectral imaging software was used to produce ratiometric images to compare emissions changes across cells and align images into three-dimensional volumes or z-stacks.

### 2.5. SEM

Stained resin blocks were coated with 10 nm carbon using a Safematic CCU-010 carbon coater. Samples were then imaged using a Tescan Mira3 Field Emission SEM (Tescan, Czech Republic) using the backscatter electron (BSE) detector at 10 kv or inside a Tescan SX8000 with the electron-beam at 2 kV and 100 pA using a secondary electron detector in Axial BSE mode. Secondary imaging was taken using a TM3000 TableTop Microscope (Hitachi, USA).

### 2.6. Image processing

Collating time-lapse light microscopy data, applying captions, changing playback speed and producing videos was achieved using Premiere Pro software (Adobe, Boston, USA). Linear changes to vector images, resampling bitmaps, improve image resolution (DPI), brightness and contrast were made using CoralDRAW graphic design suite (Ottawa, Canada) with Adobe creative cloud (Adobe, Boston, USA) to further render the images and video.

Quantitative pathology and bioimage software (Qupath, USA) [37], and ImageJ^®^ (Wisconsin, USA) quantified image data, e.g. counting cells and subcellular structures, assessing subcellular volume or surface area change in SEM micrographs.

Paired T-tests, or Wilcox *t*‐test where the data is non-normal (skewed), were applied using R-studio package 2022.02.3 + 492, to determine the significance of paired cell counts (*M. mollis n* = 8, *P. verrucosa n* = 8) and any subcellular development documented through quantitative pathology (Qupath, USA) [37] and ImageJ^®^ (Wisconsin, USA) software. Data was visualized in RStudio using the ggpubr R package.

## 3. Results

**Figure 3.**
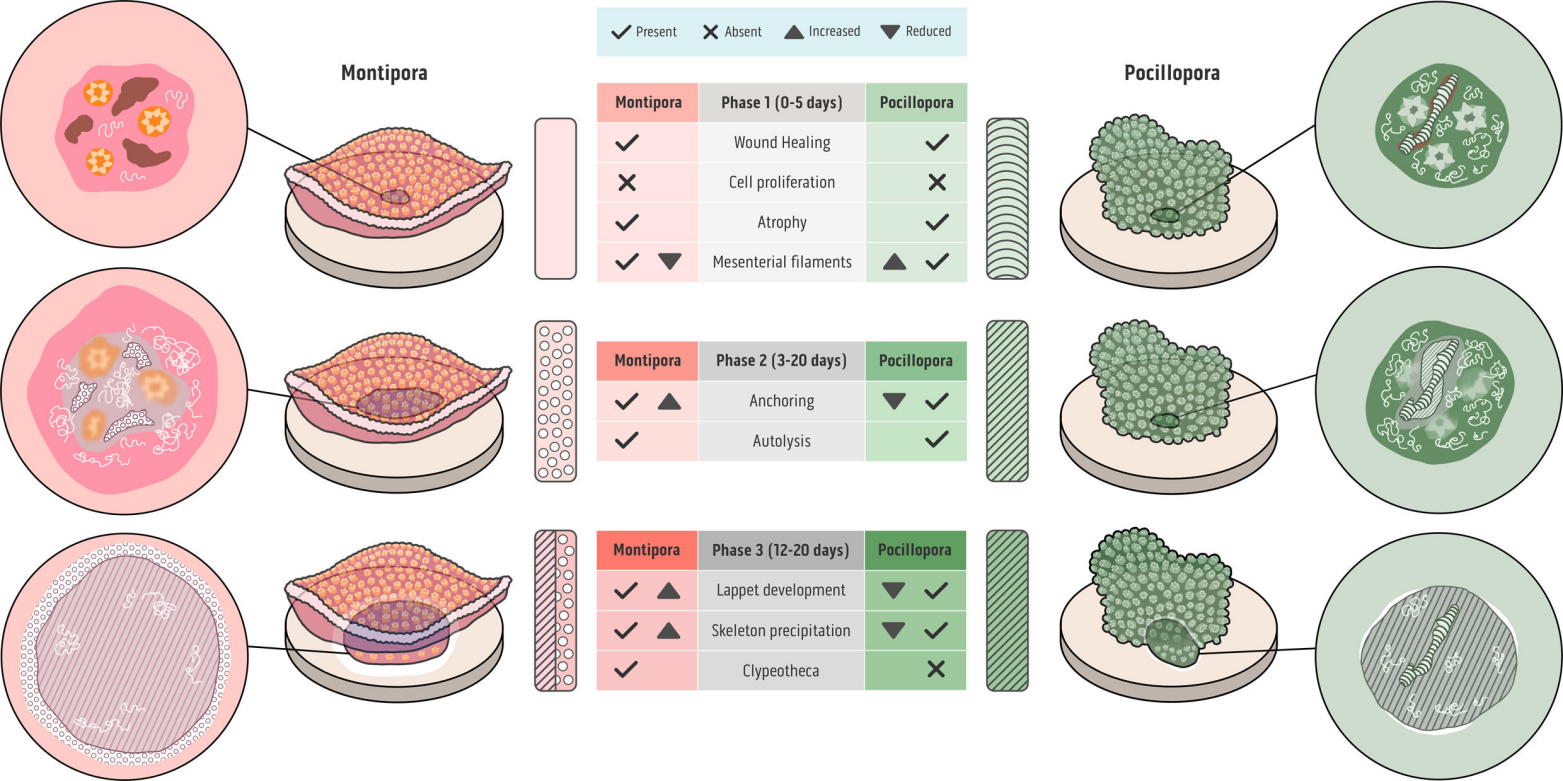
The three distinct phases for fragment attachment. In the central table, the central column shows the underlying processes for each phase and if they were comparatively more, less or equally present in the analysis for *M. mollis* (left/pink) and *Pocillopora verrucosa* (right/green). Next, the rectangle pillars in the figure represent the skeletal development at each phase: clypeotheca, stripes, secondary development and curved lines, initial contact via verrucae. A macro representation of the coral fragment on the ceramic disc follows this section. The fragment is transparent to show the underlying contact point between it and the substrate. The furthest left/right column is a magnified representative view of the contact point. Phase 1: In *M. mollis,* the contact interface showed wound healing (brown) and active mesenterial filaments (white squiggly lines) but zero skeletal development. In *Pocillopora verrucosa,* wound healing was also present at the contact interface. The mesenterial filaments (white squiggly lines) were more active in *P. verrucosa* compared with *M. mollis*. While no new skeletal development was present in either species, existing skeletal verrucae can breach the tissue layers. Phase 2: *M. mollis* and *P. verrucosa* showed signs of apoptosis and autolysis of the tissues by the mesenterial filaments, which enables the surface body wall (bright pink) to transition into a basal body wall (grey) for cell-to-substrate anchoring and skeleton development. This was not a uniform process, and as such, some fine clypeothecal accretion was present in *M. mollis*. Phase 3: Calcification and lappet-like appendage development (white rim on the basal attachment) led to bonding and encrustation. The skeleton and lappet-like appendage in *P. verrucosa* were reduced in their development compared with *M. mollis*. The skeleton microstructure in *P. verrucosa* was shingled, while in *M. mollis* it was more complex, consisting of both clypeotheca and shingled microstructures.

Asexual reproduction in the reef-building corals *Pocillopora verrucosa* and *M. mollis* adhered to the three phases of development during fragment attachment (figure 3): phase one: contact response preparing for fragment attachment (0−5 days); phase two: fragment stabilization through soft tissue anchoring (*M. mollis* 3 to 12 days and *Pocillopora* 5+ days); and phase three: lappet-like appendage development and calcification leading to permanent bonding and encrustation (12+ in *M*. *mollis* and 20+ days *P*. *verrucosa*). Each phase is represented as a range to be consistent with previous literature [1,19] and to highlight that, despite each phase sequentially initiating, the phases exhibited biological spatial and temporal variations in cellular development, mesenterial filament activity and skeletal development, resulting in varying rates of tissue growth across the contact interface. Thus, not all points of contact or tissue will develop equally depending on resource allocation, fragment health, substrate biofilm, stability and/or number and extent of tissues contacting the substrate interface. Despite this, all 3-phase responses appear to be generally conserved across taxa.

### 3.1. Phase 1: contact response of the coral fragment to the substrate

**Figure 4.**
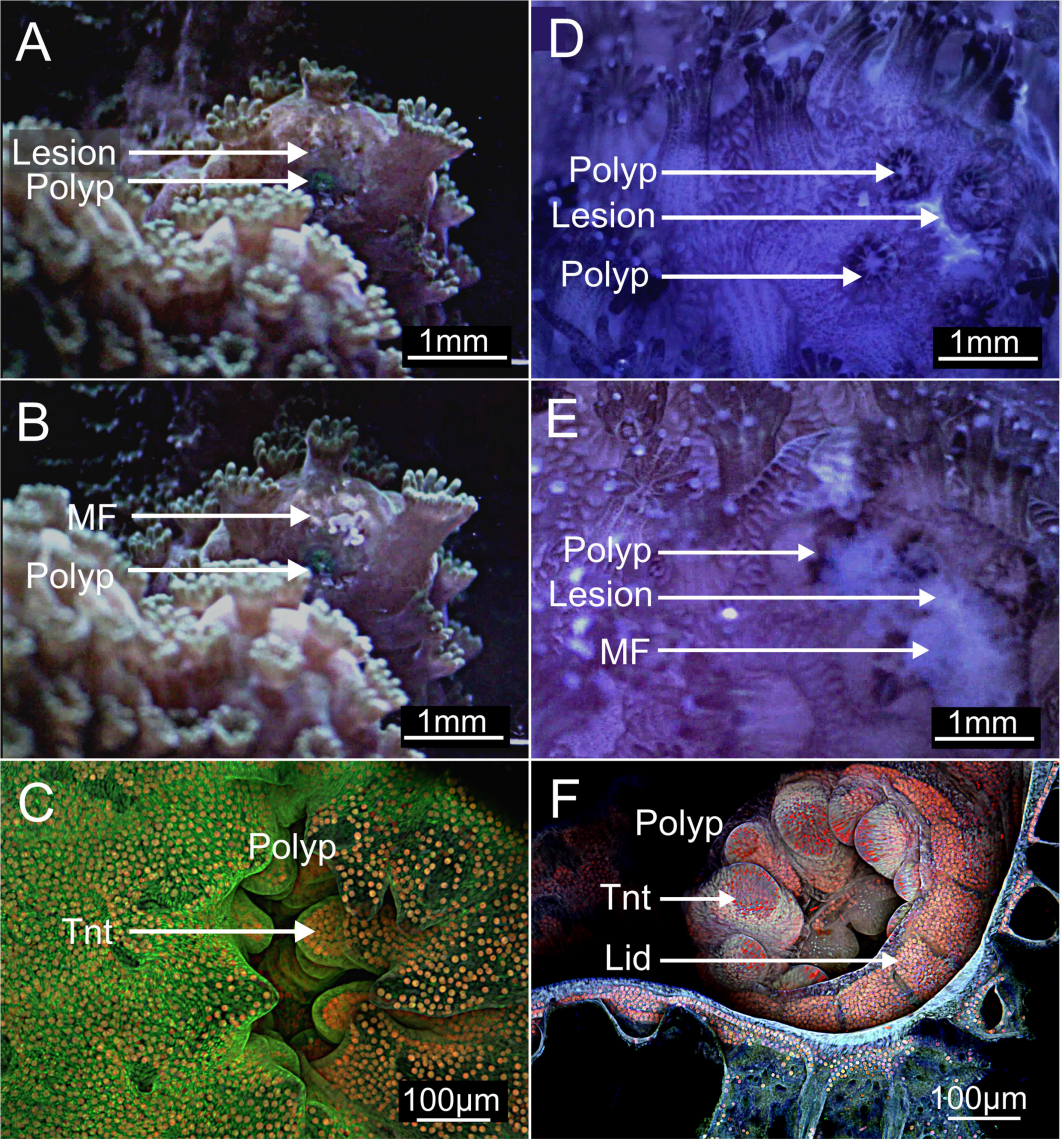
Phase 1 time-lapse optical (A,B,D,E) and confocal microscopy (C,F) detailing the behaviour of the coenosarc, mesenterial filaments and the polyp at the contact interface in *P. verrucosa* (D–F) and *Montipora* sp. (A–C). (A) and (D) Lesions formed, and the polyps retracted in the tissues at the contact interface and between the coral and the substrate in *P. verrucosa* and *M. mollis* (B) and (E). The mesenterial filaments (MF) cleaned the substrate interface and the wounds. (C) and (F) The coral polyps that come under stress at the contact interface retracted into the corallite in *M. mollis* and *P. verrucosa*; however, a tissue covering ‘lid’ (Lid) was also deployed to protect the polyp further in *P. verrucosa*. MF; mesenterial filament, Tnt; tentacle. Green and blue emission; the supporting cells and some gland cells, Green/red combined emission; *Symbiodiniaceae*, Deep red; nematocysts.

Wounds appeared as lesions at the contact–substrate interface for both *P. verrucosa* and *M. mollis* (electronic supplementary material, videos S1 and S2, figure 4). Substrate contact triggered the deployment of mesenterial filaments (filaments associated with the epidermis used in digestion with numerous granulated gland cells secreting digestive enzymes) into wounds and underlying substrate (electronic supplementary material, video S2), releasing mucus (electronic supplementary material, video S3). Tissue wounds healed in 1 to 2 days (figure 4), and the individual coral polyps that make up the colony retracted into their corallites (electronic supplementary material, video S3, figure 4A–D). The polyp in *P. verrucosa* became covered over by a retractable ‘corallite lid’ (electronic supplementary material, video S3, figure 1F). This lid structure was not present in *M. mollis* (electronic supplementary material, video S3, figure 4C).

**Figure 5.**
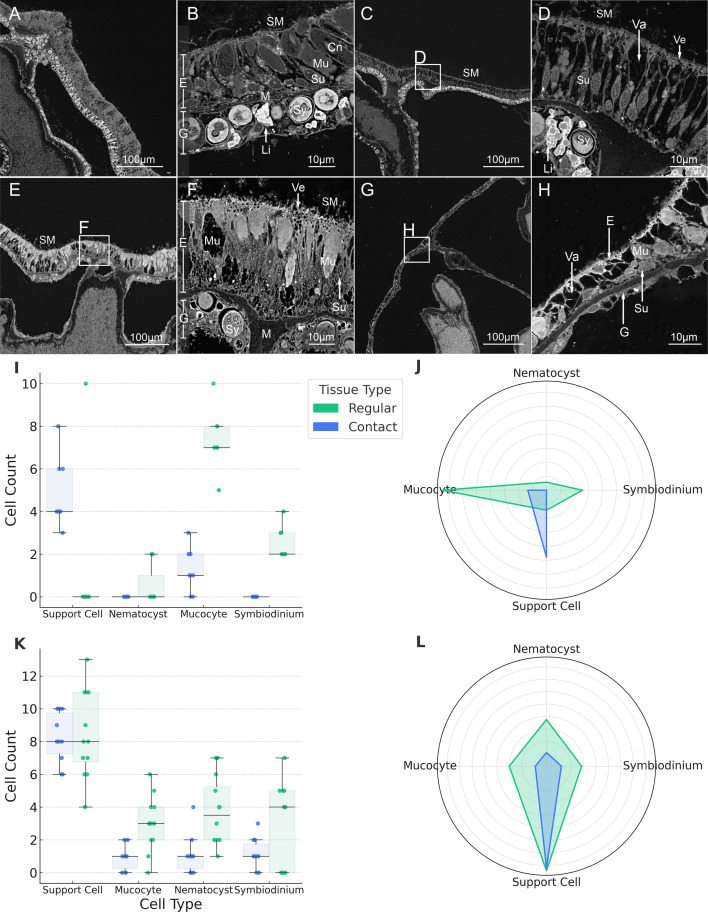
Scanning Electron Microscopy (SEM) was used to examine the fine-scale organization of surface tissues in *M. mollis* (Panels A–D) and *Pocillopora verrucosa* (Panels E–H). Healthy tissues are shown in (A,B) (*Montipora*) and (E,F) (*P. verrucosa*), while contact-affected tissues are depicted in (C,D) and (G,H), respectively. SEM imaging reveals the arrangement of the epidermis (E) and gastrodermis (G), separated by the mesoglea (M). In healthy samples (A,B,E,F), the epidermis shows a pseudo-stratified layer of supporting cells (Su), mucocytes (Mu) and cnidocytes (Cn), while the gastrodermis contains supporting cells, storage cells (Li), and *Symbiodiniaceae* (Sy). Contact-affected tissues (C,D,G,H) exhibit epidermal atrophy, with a loss of mucocytes and cnidocytes, persistence of supporting cells, and increased intracellular vacuoles (Va), particularly at the substrate interface. Box plots (Panels I and K) and radar plots (Panels J and L) demonstrate the changes in cell type composition between regular (blue) and contact (green) tissues for *P. verrucosa* (I,J) and *M. mollis* (K*,* L). These analyses reveal shifts at the tissue interface in the contact tissues, with decreased mucocytes and cnidocytes and relative retention of supporting cells.

A baseline for comparative analysis was established by first characterizing the type and differences between cell populations of the healthy surface body wall (SBW) in *M. mollis* and *P. verrucosa* (figure 5). This baseline allowed us to assess changes in cell composition at the contact interface, where tissue atrophy occurs. The healthy SBW exhibited a pseudo-stratification of cells characteristic of the coral, with the epidermis consisting of supporting cells, mucocytes (mucus-producing cells), cnidocytes (stinging cells) and gastrodermis (supporting cells, storage cells and *Symbiodiniaceae*) (figure 5A–G). These various cell types were connected via the mesoglea. These healthy tissues were compared with those at the contact interface. Wilcoxon rank-sum tests were performed to compare cell counts between contact and regular tissues for *P. verrucosa* and *M. mollis* species, using a significance threshold of *p* < 0.05.

In *P. verrucosa*, *Symbiodiniaceae* abundance was significantly reduced in contact tissue compared with healthy tissue (Wilcoxon *Z* = 0.0, *p* = 0.0003), with a large effect size (Cohen’s *d* = −4.62, 95% CI [0.30, 1.50]). Mucocyte density also showed a significant reduction in contact tissue (Wilcoxon *Z* = 0.0, *p* = 0.0009), with a similarly large effect size (Cohen’s *d* = −4.76, 95% CI [0.30, 1.50]). These reductions indicate substantial cellular atrophy (figure 5G,H). Contrastingly, we observed a significant increase in supporting cells between contact and healthy tissues (Wilcoxon *Z* = 54.0, *p* = 0.0151). However, the increase likely reflects an artefact of cellular atrophy, where the loss of mucocytes and *Symbiodiniaceae* exposes normally obscured supporting cells, making them easier to detect rather than more abundant. Nematocyst density in *P. verrucosa* did not differ significantly between tissue types (Wilcoxon *Z* = 22.5, *p* = 0.117), though the moderate effect size (Cohen’s *d* = −0.83) suggests a potential biological trend. This may simply reflect the sampling location, as cnidocytes in *P. verrucosa* are primarily concentrated around the polyp and tentacle tips, areas not targeted during SBW tissue collection.

In *M. mollis*, mucocyte density was significantly lower in contact tissue compared with healthy tissue (Wilcoxon *Z* = 15.0, *p* = 0.0028), with a large effect size (Cohen’s *d* = −1.64). Nematocyst abundance also differed significantly between tissue types (Wilcoxon *Z* = 12.5, *p* = 0.0016), with a similarly large effect size (Cohen’s *d* = −1.55), indicating strong tissue-level responses similar to *P. verrucosa*. By contrast, no significant differences were observed in support cell abundance (Wilcoxon *Z* = 57.5, *p* = 0.894; Cohen’s *d* = −0.10) or *Symbiodiniaceae* density (Wilcoxon *Z* = 45.0, *p* = 0.327; Cohen’s *d* = −0.83). *Symbiodiniaceae* in the gastrodermis of *M. mollis* were irregularly distributed throughout the gastrodermis, with a high number of cells in some cross-sections but not in others (figure 5I). Like in *P. verrucosa*, support cells in the contact tissue of *M. mollis* appeared thinner and exhibited prominent rounded vacuoles at the apical surface (figure 5). The presence of these vacuoles in contact tissues of both species suggests they may remain active in secreting a biofilm or ingesting biofilm-associated materials to aid in colony protection. Phase 1 ended when the contact tissues began anchoring to the contact surface.

### 3.2. Phase 2: soft tissue anchoring and fragment stabilization

**Figure 6.**
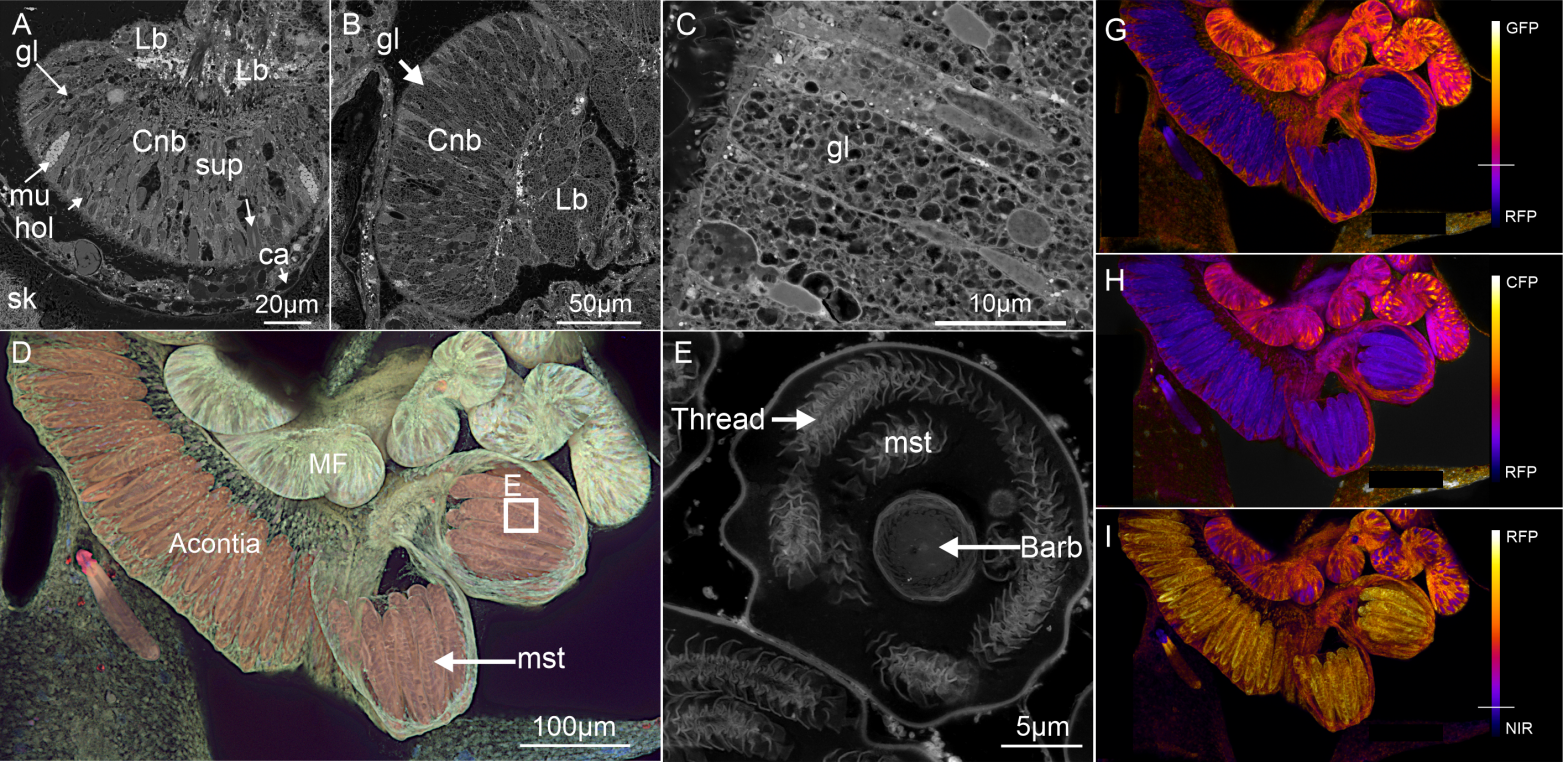
Phase 2 Scanning Electron Microscopy (A–C*,* E) and Confocal Laser Scanning Microscopy *(*D, G–I) with intensity profiles (G–I) of auto-fluorescence in *M. mollis* mesenterial filaments (MFs). MFS possess three cellular arrangements: (A) Type I arrangement consisting primarily of supporting cells (sup), auto-fluorescent gland cells (mu and gl) and medium (approx. 20−50 µm) nematocysts (hol). (B) and (C) Type ii bi-lobed (Lb) mesenterial filament consisting of cnidoglandular band (Cnd) and lobes possessing a unicellular population of unidentified gland cells (gl) with the occasional nematocyst. (D) and (E) Type iii mesenterial filament (acontia) with a unicellular population of large (100–120 nm) nematocysts resembling *P mastigophores* (mst) arranged as a battery. The large mastigophores emitted auto-fluorescence in the red wavelength in the type iii filament but not the type i. Paired auto-fluorescence emission ratios of type iii mesenterial filament showing the dominance of the red emission (RFP) to compare with (G) green (GFP), (H) blue (CFP) and (I) red (NIR) emissions.

**Figure 7.**
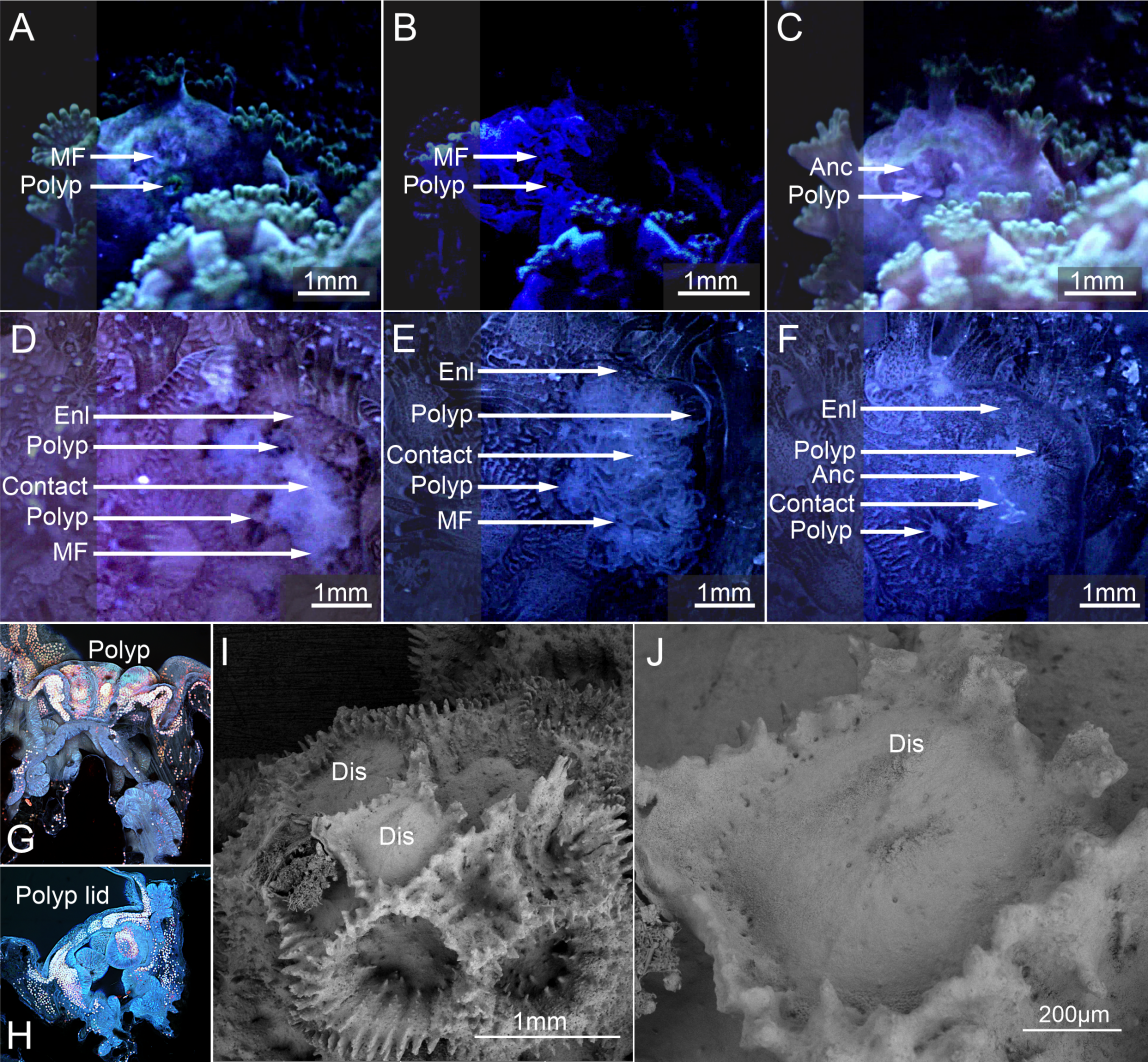
Phase 2 time-lapse optical (A–F) and confocal (G,H) and Scanning Electron Microscopy (SEM) describing the behaviour of the tissue at the contact interface (I,J). (A) and (D) The soft tissues in contact (contact) with the substrate adapt, expand (Enl) and spread onto the substrate in *M. mollis* (A–C) and *P. verrucosa* (D– F). The fragment then deploys mesenterial (MF) filaments for autolysis of the redundant tissue and polyps (Polyp) (B) and (E), leaving a transparent contact surface. (C) and (F) As a result of autolysis, new cells form that allow the tissues to become attached/anchored (Anc), creating a contact interface sealed off from the surrounding water column. (F) In *P. verrucosa,* the coral’s existing skeleton is present at the contact (Contact). (G) and (H) Autofluorescence shows the polyps at varying phases of degradation. (I) and (J) SEM show the polyps' corallite is closed off by shallow forming dissepiments (Dis).

After wound recovery, soft tissues expanded and spread onto the substrate in both *M. mollis* and *P. verrucosa* (figure 3, electronic supplementary material, videos S4 and S5), which resulted in a soft tissue anchor/attachment. Soft tissues of both species moulded to the substrate depth profile, increasing the contact surface area and anchored surface (figure 6). Autolysis (the self-digestion of the cell/tissues) was triggered (figure 7, electronic supplementary material, videos S4 and S5), accelerating the removal of polyps and cells in the SBW to leave the contact tissue transparent in optical images (figure 7).

**Figure 8.**
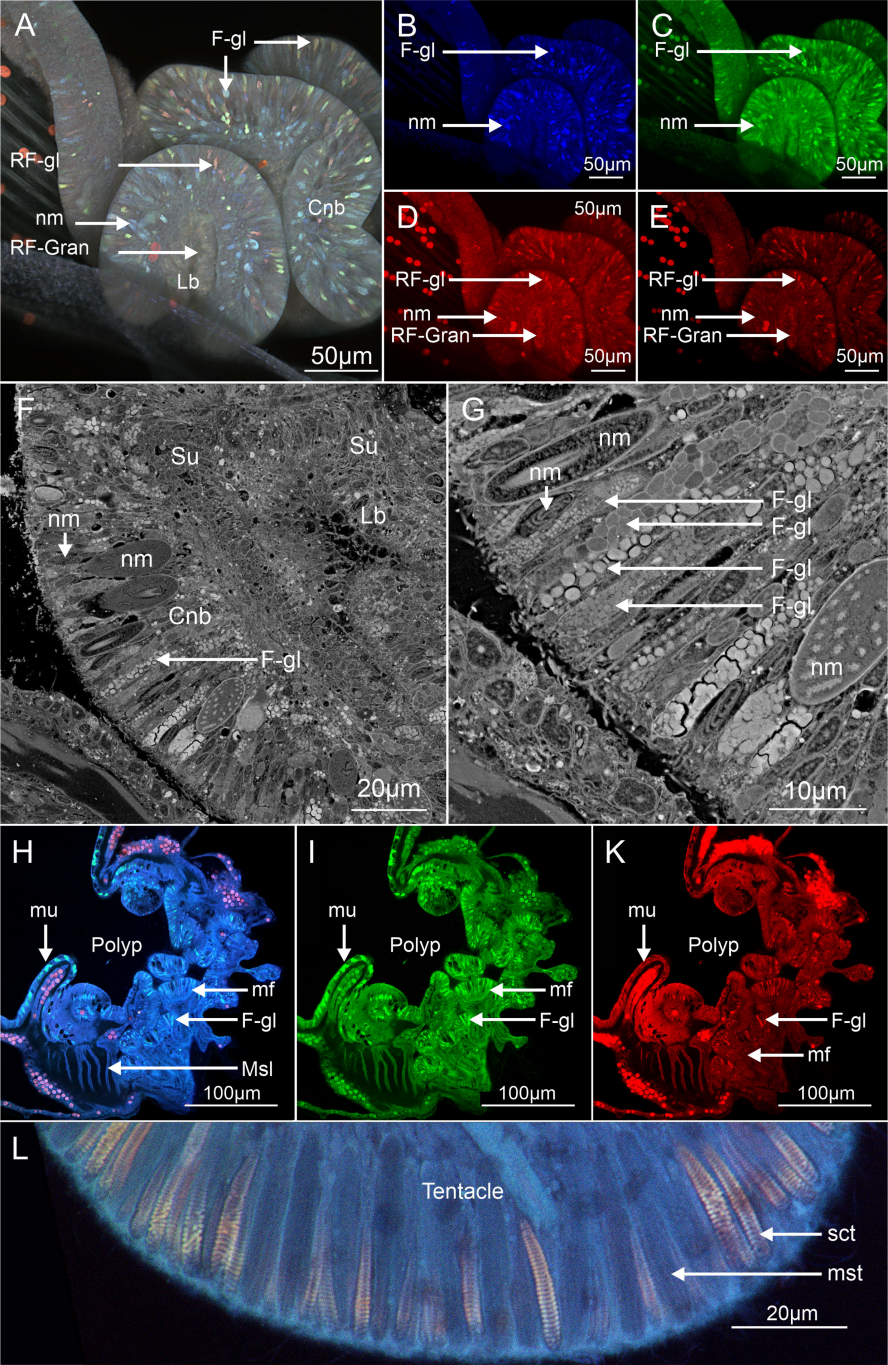
Phase 2 Confocal Laser Scanning Microscopy Scanning Electron Microscopy showing auto-fluorescence of the mesenterial filaments (MFs) and in *P. verrucosa* (A) A z-stack of MFs auto-fluorescence in *P. verrucosa* with Type i morphology located in the Cnidoglandular band (Cnb) were auto-fluorescent cells, including Nematocysts (nm) and gland cells (RF-gl and F-gl) in the digestive lobes with granular material (RF-Gran) (Lb). (B to E) Autofluorescence from the stacked image (A) in blue, green, red and deep red wavelengths. Mesenterial filaments (MFs) excited by blue (B) and green (C) wavelengths, the nematocysts and the granular gland cells in the MFs emitted auto-fluorescence in red and green. No auto-fluorescence from nematocysts when excited by red (D) and deep red (E); however, some gland cells (f-gl) in the cnidoglandular band and the granular material (RF-Gran) in the lobes did emit deep red, noticeable in (A). (F and G) SEM show the cell structure of the MFs. Highlighting the supporting cells (su), the array of cnidocytes (nm) and gland cells (unidentified) that characterize a type i MFs. (H to K) Red and deep red emission is present in some gland cells in the MFs and not present in the gland cells (mu) of the epidermis; instead, these cells emit red (A) and green (B). (I) Two types of nematocysts were observed in the polyp tentacles (mst and sct). The sct strongly emitted in red and deep red (I) while the mst had limited signal.

Mesenterial filaments in *M. mollis* possessed three distinct morphologies with different cellular arrangements (or ‘types’); (i) consisting primarily of supporting cells, gland cells and medium (approx. 20−50 µm) cnidocytes (*holotrichs*) (figure 6A), (ii) consisting mainly of an unknown gland cell with rare scatterings of nematocysts (figure 6B) and (iii) large (100–120 µm) cnidocytes (*P mastigophores*) arranged as a an linear array or battery (figure 6D). Conversely, the mesenterial filament of *P. verrucosa* possessed only a single morphology type (i) (figure 8A), although they were more active.

The cnidoglandular band (bulbous lobes) of the mesenterial filaments in *P. verrucosa* possessed previously uncharacterized gland cells (figure 8B–E). The gland cells appeared to possess different subcellular vesicles in SEM (figure 8F,G). In CLSM, the vesicles emitted at wavelengths associated with cyan fluorescent proteins (CFP; 425 to 475 nm) and green fluorescent proteins (GFP; 500 to 550 nm) when excited by blue (405 nm) and green (488 nm) wavelengths (figure 8B,C). Gland cells in the cnidoglandular band emitted at the same wavelength as the mucocytes observed in the *P. verrucosa* coenosarc (tissue covering the skeleton between polyps) and polyps, indicating these cells may have similar functions.

Neither the gland cells nor mucocytes emitted when excited by red (561 nm) and near-infrared (640 nm) wavelengths. However, one uncharacterized gland cell did emit red fluorescence when excited by red (figure 8D) and deep red (figure 8E) (RFP; 500–620 nm, NIR, 663–738 nm). Granular material in the mesenterial filaments digestive lobe, adjacent to the cnidoglandular band, also emitted red and deep-red wavelengths. The granules also emitted in green (CFP—425 to 475 nm), giving them a unique autofluorescence profile compared with the gland cell vesicles of the cnidoglandular band (figure 8H,I).

In CSLM, the *M. mollis* large mastigophores (barbs) that were present in the type iii mesenterial filament cnidocytes emitted red auto-fluorescence (figure 6D*,*G–I). No red emission was observed in the mastigophores in *P. verrucosa*. However, in *P. verrucosa*, a red and deep red emission was present in the spiral thread of the spirocysts (spiralled barb) located in the tip of the polyp (figure 6L). Phase two of the attachment process ended as the soft tissue anchor developed a lappet-like appendage at the rim of the tissue and skeletal precipitation began.

### 3.3. Phase 3: skeleton precipitation at the contact interface and encrustation of the substrate via the lappet-like appendage

**Figure 9.**
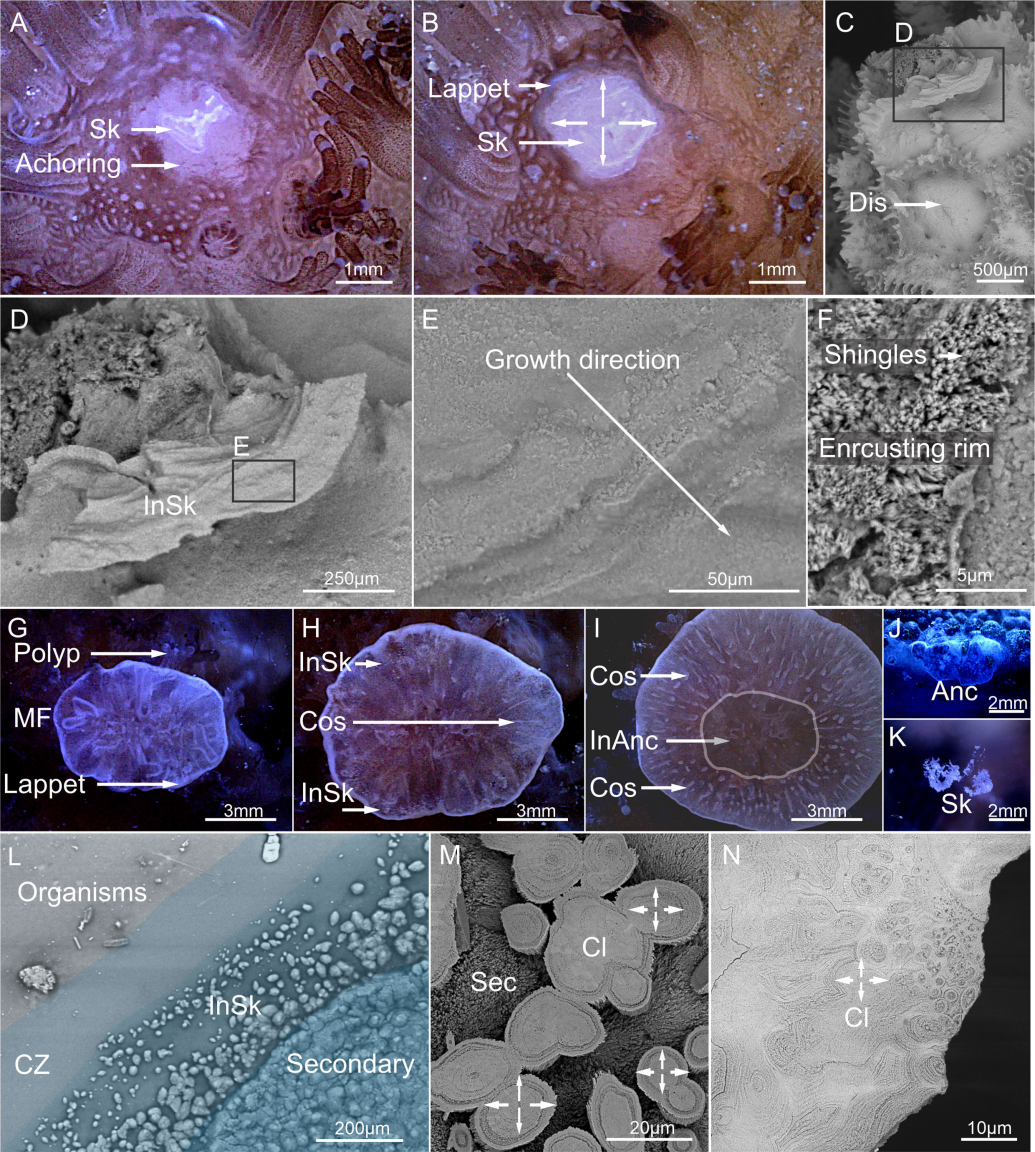
Phase 3 time-lapse optical, confocal and electron microscopy of the contact interface between the coral and substrate. Skeleton growth in *P. verrucosa* (A,B) started from a single contact point (Sk) and radiated outward (C–E), with a shingled microstructure (F). (C) Where polyps were removed during Phase 2, the defect corallites were sealed over by shallow-forming dissepiments. (G,H) The soft tissue anchor in *M. mollis* eventually developed into a full soft tissue attachment. (I) This attachment (InAnc) began encrusting over the substrate surface once a lappet-like appendage formed, producing both an initial layer of skeleton (InSk) and secondary development ([cos] costae and thickening). Mesenterial filaments (MF) were active at the micro interface throughout the encrustation process. (L–N) Unlike in *P. verrucosa,* the initial layer of skeleton growth in *M. mollis* started from multiple contact points, radiating outwards in a similar way to clypeotheca (InSK and Cl). (I) The initial layer was thickened by secondary shingled skeleton growth (M,N). There was a distinct ‘clean-zone’ (CZ) L in *M. mollis.*

**Figure 10.**
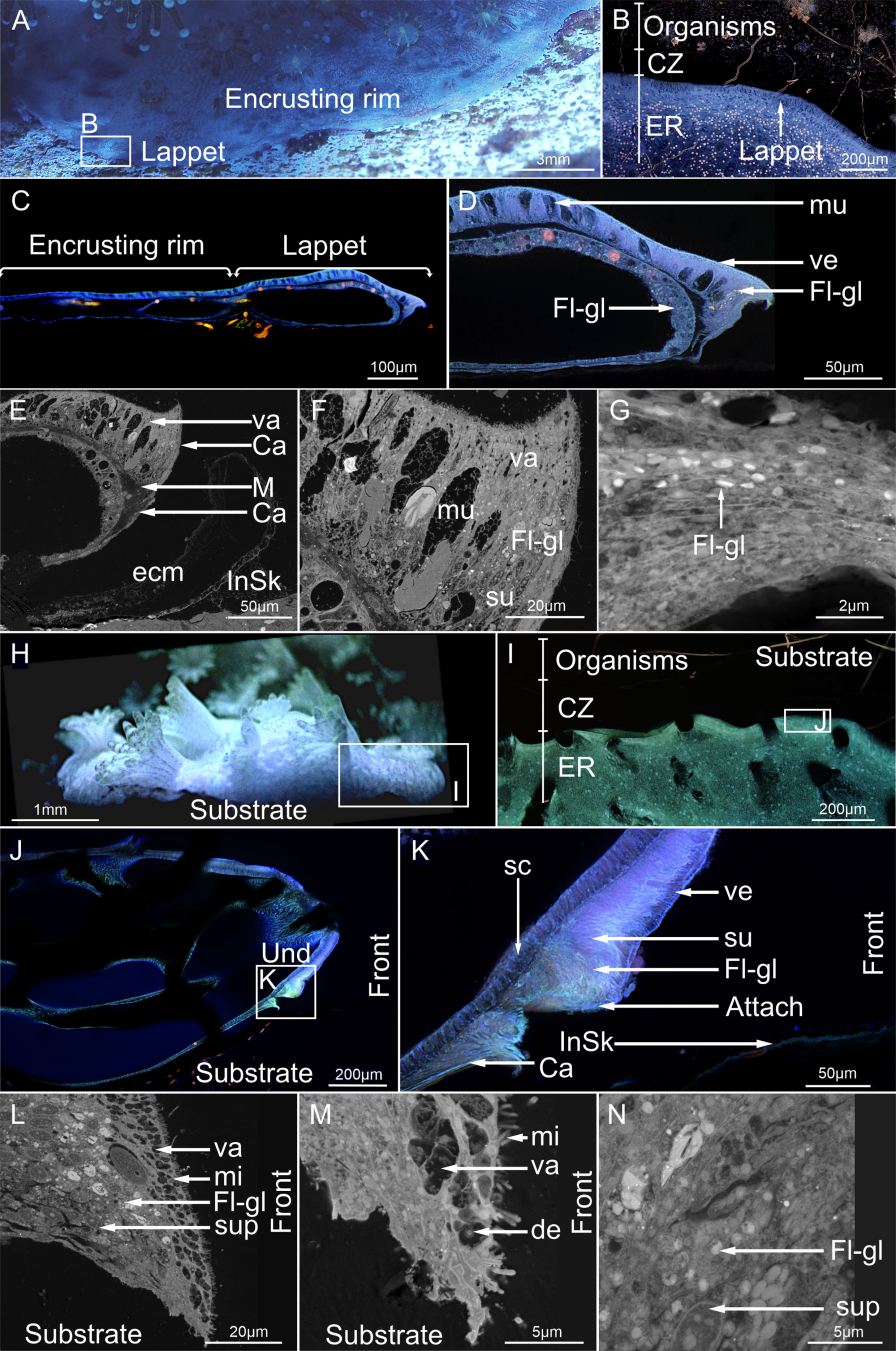
Phase 3 time-lapse optical, confocal (CLSM) and Scanning Electron Microscopy (SEM) of the lappet-like appendage and skeletal microstructure. Lappet-like appendage in *P. verrucosa* (A,B) was thin and appeared as a tissue ridge at the edge of the encrusting rim. The substrate in front of the lappet-like appendage (B) is an area (CZ) of reduced fluorescence. (C–G) There is a separation between the lappet-like appendage and the skeleton/or substrate, which was caused by sample preparation. Red-fluorescing pigmented granular vesicles (Fl-gl) were close to the substrate. The same/similar fluorescent granules were present in the gastrodermis (D). (E). The calicoderm (Ca), which produced the extracellular matrix (ecm) for skeletal precipitation (InSk) developed under or as part of the epidermis, facilitated by a splitting of the mesoglea (M). (E–F) The cells in the lappet-like appendage in *P. verrucosa* were primarily non-fluorescent mucocytes with vacuoles (secreted mucus) (D). Supporting cells were difficult to observe because of their small size, but were condensed at the ‘hooked’ tip closest to the substrate (G) and (D) associated with fluorescent granules (Fl-gl). Supporting cells possessed small vacuoles at the surface of the cell (F) and (D), which did not fluoresce. (H). The lappet-like appendage in *M. mollis* was thicker and located at the same location as a clean zone (CZ). The CZ was not as prominent in fluorescence compared with *P. verrucosa,* but was easily observed when correlated with SEM (figure 10). Like *P. verrucosa,* the area attached (attach) to the substrate possessed a higher concentration of red-fluorescing (Fl-gl) granules. (L,M) Almost no mucocytes were present in *the M. mollis* lappet-like appendage, which primarily consisted of supporting cells with large vacuoles (va) filled with debris (de), located at the distal tip of the cell*,* contacting the substrate. (N) Higher concentration of red-fluorescing granules was found closer to the substrate in supporting cells.

Once autolysis stopped and the basal body wall (BBW) formed, the skeleton began to precipitate at the interface between the anchored tissue and substrate (figure 9). A lappet-like appendage developed at the rim of the anchored soft tissues in both species to facilitate the forward extension of the anchored tissues and skeleton over the substrate forming a basal attachment (electronic supplementary material, videos S6 and S7, figures 9 and 10). The formation of lappet-like appendages took approximately up to 36 days in *P. verrucosa*, whereas in *M. mollis*, it occurred in about 12 days.

The soft tissue anchor in *M. mollis* eventually developed into a full soft tissue attachment (figure 9), which began growing over the substrate surface, producing a basal skeleton and forming an encrusting rim once a lappet-like appendage formed (figure 10B–I). The encrusting rim and skeletal growth (both initial and secondary) in *P. verrucosa* were thinner and developed more slowly than in *M. mollis* (figure 9). Skeleton growth in *P. verrucosa* started from a single contact point radiating outward, surrounding the preexisting skeleton protruding through the tissues (figure 9B–E). This new growth in *P. verrucosa* appeared to have a shingled microstructure like the rest of the skeleton (figure 9F). Defect corallites caused by polyp removal or covering by the polyp lid during Phase 2 were sealed over by shallow-forming dissepiments (figure 9C). Like *P. verrucosa,* the initial precipitated skeleton layer from the lappet-like appendage in *M. mollis* had radiating growth (figure 9). Unlike in *P. verrucosa*, the initial layer of skeleton growth in *M. mollis* formed from multiple points, developing as heterogeneous, non-shingled, centripetal growth. This growth radiated from the calicoblasts in the BBW and the calicodermal extension underlying the lappet-like appendage and sitting between the lappet and the substrate/skeleton (figure 10). The centripetal growth eventually converged, forming a solid layer (figure 9L–N), resembling clypeotheca (a structure that develops over the exposed coral skeleton following stress or damage). In *P. verrucosa,* secondary skeleton growth by the BBW trailing the lappet-like appendage was reduced or absent, lacking costae (rib-like) walls, unlike *M. mollis* (figure 9D,I).

Lappet-like appendage morphology differed between *M. mollis* and *P. verrucosa* (figure 10). The appendage was larger in *M. mollis* (approx. 600 µm thick and approx. 150 µm, respectively; figure 10) and produced an independent underlying calicoblast extension. When fully extended, the undulated tissue in both species appeared like a hooked tip (figure 10E,I), hooking around the skeleton as the new skeleton was being precipitated, sealing the area from the surrounding environment and producing an extracellular matrix for skeletal production. Both species exhibited a ‘clean zone’ in front of the lappet-like appendage (figure 10I,B).

Cells present in the lappet-like appendage varied between *M. mollis* and *P. verrucosa* (figure 10). Both the gastrodermis and epidermis (SBW) of the lappet-like appendage in *M. mollis* were densely packed with storage and supporting cells, respectively (figure 10K). The epidermis of *P. verrucosa* consisted of multiple cells, including goblet-shaped gland cells and supporting cells (figure 10D–E). All cells in *M. mollis* and *P. verrucosa* were pseudostratified, with the supporting cells developing more significantly towards the ‘base’ or underside of the lappet-like appendage epidermis (figure 10F,K). The supporting cells for both *M. mollis* and *P. verrucosa* developed vesicles that emitted in red and green wavelengths (figure 10D,K), similar to the gland cells in the mesenterial filaments of *P. verrucosa* (figure 8). The supporting cells of *M. mollis* possessed non-fluorescent vacuoles concentrated at the apical surface of the cell (figure 10M). The vacuoles contained debris potentially ingested through endocytosis. Similar, smaller vacuoles were found in the lappet-like appendage in *P. verrucosa* (figure 10D,F). The smaller vacuoles correlated with the development of fluorescent vacuoles in both *M. mollis* and *P. verrucosa*.

## 4. Discussion

*M. mollis* and *P. verrucosa* exhibited a three-step CAM process similar to that observed in *A. millepora* [1] (figure 3), suggesting similar processes are driving attachment in reef-building corals and are crucial for success. Improved heavy metal staining, dehydration and embedding techniques used for optical and SEM imaging applied in our current study resulted in better preservation of microstructures and autofluorescence signals, facilitating higher-resolution imaging to observe novel developmental features and structures during the attachment process. While this three-step development process was broadly conserved across taxa, we observed taxonomically distinct variation in immune response, behaviour, tissue development and skeletal microstructure during each phase (summarized in figure 3), which likely accounts for the observed disparities in attachment rates, robustness and efficacy among species [19,20]. Understanding attachment not only enhances our strategies for coral restoration but also sheds light on the evolution of colonial growth forms and asexual reproduction mechanisms.

### 4.1. Phase 1 and Phase 2: first contact and soft tissue anchoring

#### 4.1.1. Cellular development at first contact

In Phase 1, *M*. *mollis* and *P. verrucosa* underwent wound healing, mucus release and a degeneration of the tissues, aiding in safeguarding the fragment while contacting a foreign substrate [38,39] (figure 3). *A. millepora* undergoes cell proliferation (instead of cell atrophy), increasing musculature that likely facilitates a faster transition to Phase 2 (figure 3). However, cell atrophy can also be beneficial, reducing metabolic demands on affected tissues and helping to remove damaged or infected cells [40,41].

Autolysis typically refers to the self-digestion of cells after death and plays a crucial role in tissue turnover, development and the removal of damaged or unwanted cells [42,43]. As a result, autolysis can improve local metabolism, enhancing tissue recovery, remodelling and rapid tissue growth needed for anchoring and basal encrustation [1]. Cell autolysis is typically carried out by immune cells, such as macrophages or gland cells [44]. While direct observations of cell changes in type and abundance, combined with tissue removal by the mesenterial filaments, suggest autolysis is common during the attachment process [1], morphological analysis alone cannot confirm the mode of autolysis. As such, further molecular or metabolic analysis is needed to determine the mesenterial filaments and/or the cell function.

#### 4.1.2. Mesenterial filaments

Like most coral tissues, mesenterial filaments in reef-building corals are understudied [45], with most current knowledge extrapolated from studies on sea anemones, leading to limited understanding of cell ultrastructure and function. Here, we observed that mesenterial filaments were continuously active throughout all phases of attachment for *M. mollis* and *P. verrucosa*, similar to *A. millepora* [1] indicating a significant and ongoing role in preparing the substrate and coral tissues for attachment.

The improved sample processing facilitated the identification of three types of mesenterial filaments, two of which were previously unreported. According to Kinchington (D Kinchington 1980, unpublished data), the filaments were classified into three mesenterial filament types based on the different cell types present. Type I is functionally a multifaceted structure, aiding in digestion, secretion and partially colony defence, demonstrated by the high number of gland cells and supporting cells, the presence of cnidocytes. Type II features large, irregular gland cells whose microstructure was vacuolated, indicative of a continuous secretion of materials such as mucus. Type III (acontia are located at the distal end of the filament and are composed entirely of P-mastigophores, suggesting a specialized role in colony defence and competitive aggression, similar to Type II filaments. Observed presence in our study expands the only known occurrence of acontia in coral beyond the deep-water *Lophelia pertusa*, and highlights parallels with sea anemones, where acontia morphology serves as a key taxonomic trait due to their morphological diversity [45–47].

The discovery and characterization of acontia and numerous types of mesenterial filaments in our study could indicate that coral filaments serve similarly broad functional roles to those described in sea anemones [45]. For example, in anemones, mesenterial filaments and acontia contribute to immune defence, wound healing and mucus secretion [48–50] supporting tissue recovery and adaptation to stress. Similarly, our study, and prior investigations into coral attachment [51], have observed filaments active at the tissue–substrate interface, particularly in wounded or stressed regions. These results reinforce the emerging view [1] that mesenterial filaments play a more significant role in coral resilience and recovery than previously recognized, a role likely underreported due to limited investigation. Further targeted research is needed to uncover the full scope of these functions, particularly given their potential significance in coral resilience and stress response.

#### 4.1.3. Tissue composition at the contact interface

In *P. verrucosa*, we observed a retractable tissue or ‘lid’ covering the polyp, which created a temporary protective layer and seal during stress, such as contact with the substrate. The lid structure, which has not been observed previously in coral, acts similarly to an eyelid or soft tissue operculum [52]. Beneath the lid, tissues form a pseudo-operculum-like skeletal dissepiment, similar to those observed in fossilized rugose and tabulate corals that have been inundated by sediments [53] indicating that the lid structure may have also aided dissepiment formation for more permanent colony protection. While important to fragment survival, this tissue covering and rapid dissepiment growth step was not present in *M. mollis,* which may be one varying factor that controls the differences in attachment speeds.

#### 4.1.4. Anchoring

In Phase 2, all three species underwent soft tissue anchoring and subsequent cell removal through autolysis, which can be easily interrupted via physical disturbance [1]. However, anchoring tissues exhibited reduced cellular diversity and musculature (no obvious myofibril increases, unlike in *A. millipora* [1]) in *M. mollis*, and *P. verrucosa*, in comparison with *A. millepora*, thereby reducing the capacity for cell-to-substrate adhesion [1,54,55], which could indicate a reduced initial attachment strength or stability. As such, initial attachment may not be as successful without lows in environmental energy or support such as zip ties, CoralClip® or natural interlocking with the reef substrate.

### 4.2. Phase 3: encrustation for robust attachment

#### 4.2.1. The lappet-like attachment appendage

We observed the lappet-like appendage, previously described only in *Acropora millepora* [1], for the first time in *P. verrucosa* and *M. mollis*. The term ‘lappet-like appendage’ was first introduced by Lewis *et al*. [1] to describe a structure resembling the lappet of epithecate corals in its morphology and position at the colony edge. However, their functions differ. The lappet at the outer margin of the body wall secretes calcium carbonate to form the epithecal wall, thereby contributing to vertical skeletal wall growth around the corallite. By contrast, the lappet-like appendage extends laterally across the substrate, indicating a distinct role in attachment rather than upward extension [56,57]. To reflect this functional distinction, we hereafter refer to the lappet-like structure in scleractinian corals as the ‘attachment appendage’.

#### 4.2.2. Taxonomic variations in the attachment appendage

The attachment appendage in *M. mollis* and *A. millepora* appeared more effective at securing colonies to the substrate, facilitating faster attachment and the formation of a thicker, more structurally complex skeleton than *P. verrucosa*. Such enhanced skeletal development and accelerated growth may be linked to a distinct population of cells located at the distal edge of the appendage, where it contacts the substrate or interacts with potential competitors during encrustation [1,26]. These uncharacterized cells possessed vacuoles partially filled with broken-down debris, resembling cells undergoing phagocytosis or, more likely, endocytosis [58]. This evidence of phago/endocytosis, coupled with the high degree of localized storage cell development, suggests enhanced localized digestion and energy acquisition at the distal edge of the attachment appendage [58–60]. This digestion and subsequent energy acquisition at the substrate interface may explain the formation of clean zones ahead of the attachment appendage, similar to cleaning by the mesenterial filaments [1,51], where cells actively remove barriers, pathogens and competitors [26]. Clearing likely creates a safe area for rapid tissue and skeletal expansion [26], while localized nutrient uptake provides the energetic resources necessary to support increased skeletal deposition and appendage growth. Together, these processes may explain the more complex skeletal attachment [61,62] and the larger, more structurally complex lappet observed in *M. mollis* and *A. millepora*, compared with *P. verrucosa*, which lacked these same specialized cells [26].

Histological analysis of the attachment appendage in *P. verrucosa* revealed a large volume of mucocytes concentrated at the distal edge of the attachment appendage instead of phago/endocytic cells observed in *M. mollis* and *A. millepora*. These mucocytes appeared vacuolated, with newly formed, non-vacuolated cells developing at their base, suggesting active secretion and high cellular turnover. Given their abundance and position, the mucocytes, like the phago/endocytic cells, may contribute to the formation of clean zones by clearing surface debris and potential competitors [1,26,51]. However, the metabolic investment involved in producing the mucus likely imposes a significant energetic cost [63–65] diverting resources away from calcification. This may explain the reduced skeletal development and smaller attachment appendage observed in *P. verrucosa*, reflecting a clear trade-off between mucus production as part of the immune response and structural development [26]. The reduced skeletal thickness and complexity of *P. verrucosa* prompt critical questions about its ability to withstand physical stress during early developmental stages. Unfortunately, no literature to date has tested detachment thresholds in coral species, and current methods that rely on basal growth to define attachment robustness or success in coral fragment outplants [19,66,67] may yield misleading or inconsistent results across species. We recommend sheer strength testing of basal development to address this gap, as no studies to date have quantified detachment metrics in coral species [68].

#### 4.2.3. Taxonomic similarities in the coral skeleton

Taxonomic similarities in coral skeletal micro/ultrastructure that repeat to constrict macro skeletal structure, such as clypeotheca and costae, may suggest close evolutionary relationships [69–71]. Coral microstructural patterns are highly conserved and have often informed phylogenetic inference in the absence of genetic data [69,71]. In particular, distinct patterns of crystal arrangement in skeletal thickening deposits are governed by cellular processes. As such, both skeletal microstructures, particularly those forming the basal layer, and the associated soft tissues involved in attachment (e.g. the attachment appendage) may be consistent across closely related taxa. For example, in *M. mollis* and *A. millepora,* both species of the family Acroporiidae have a distinctive flattened ‘shingle’ microstructure conserved for over 40 million years. Meaning comparable formation of clypeotheca, which is followed by secondary thickening development consisting of a shingled layer and the septa/costae, which occurs for both species. Yet, in the family Pocilloporidae (here represented by *P. verrucosa*), the clypeotheca (along with costae) is absent, leaving only a layer of subsequently formed, relatively equidimensional bundles (comparable with shingles) of skeleton finer than both *M. mollis* and *A. millepora*.

#### 4.2.4. The evolutionary importance of the attachment appendage in *Scleractinia*

The shift from the lappet (epithecate forms) to the attachment appendage represents a key morphological innovation in the emergence of reef-building corals, as it enables (i) stronger basal attachment, providing greater stability in high-energy environments, (ii) increased integrated coloniality through larger colony sizes, and (iii) facilitates asexual reproduction [1,57,72]. Previously, the emergence of colonial reef-building in *Scleractinia* has been primarily attributed to the development of the coenosarc, the connective tissue between polyps, which enabled integrated colonial growth and resource sharing [56,73]. The coenosarc (reef-building forms) is theorized to have emerged from the edge-zone tissue (epithecate forms), which, like the coenosarc, serves as the connective tissue between the polyp and the lappet [74]. Interestingly, the lappet and the attachment appendage not only share similar skeletal development (epitheca and clypeotheca), cell populations and morphology, but are also both positioned at the distal-most edge of the edge-zone and coenosarc tissues, respectively [1,57]. The adaptation of the coenosarc may have occurred in tandem with the adaptation of the attachment appendage, resulting in the ability to expand growth across the substrate via the attachment appendage, rather than vertically, as in the lappet appendage, thereby enabling (i) improved stability in high-energy environments, (ii) increased integrated coloniality through larger colony sizes, and (iii) asexual reproduction [56,73]. All three factors were critical for the emergence of reef-building corals, particularly in high-energy reef environments [73].

#### 4.2.5. Limitations in cellular taxonomy

Despite decades of coral research and the advancements presented in this study, the cellular structure and function underpinning attachment and other fundamental coral processes remain underexplored, with much of the field still grounded in seminal studies that, while foundational, have seen limited advancement through applying new research technologies [27,31,75]. Future research could address this gap by combining three-dimensional tissue characterization via volume electron microscopy (vEM) with high-resolution molecular, single-cell or metabolomic approaches to reveal the organization and activity of distinct cell types. Integrating these advanced approaches with the microscopy techniques used in this study may help resolve the identity, function and spatial dynamics of the cell types involved in attachment, particularly those active during tissue degeneration, immune response and skeletal initiation. This deeper resolution could significantly advance coral cell biology by revealing how structural and metabolic processes vary across species, life stages and environmental contexts. There are implications of attachment mode for natural reef recovery and coral restoration.

#### 4.2.6. Selective coral outplanting

Species-level variation in coral attachment underscores the need for an eco-evolutionary approach to reef restoration, one that selects outplant species based on key taxonomic traits and environmental compatibility [76,77]. At present, outplant success is too often evaluated *post hoc*, following opportunistic deployments, rather than through trait-informed planning conducted *a priori*. Trait-based strategies help practitioners determine which species to plant, where, and when, increasing the likelihood that fragments reach self-sustaining attachment before seasonal storms or other disturbances can dislodge them[19,20,77]. This is particularly relevant for species like *P. verrucosa*, which develop less complex skeletal structures and may take longer to achieve stable, self-sustaining attachment (Suggett, pers. obs..)—despite exhibiting similar basal growth rates to other species. However, what constitutes self-sustaining attachment and the strength thresholds required to resist dislodgement remains undefined, limiting our ability to assess when restoration efforts have truly succeeded [68].

To begin resolving this, the development of self-sustaining attachment must be quantified across time and taxa. This includes assessing whether variation in the developmental biology of the three attachment phases correlates with measurable increases in attachment strength [68]. One approach is to track the progression of basal skeletal development across species and compare it against hydrodynamic forces typical of ambient and elevated reef conditions. Aligning internal structural development with external force thresholds would allow us to pinpoint the moment when a fragment becomes mechanically self-sufficient, i.e. has the capacity to resist dislodgement without artificial support. Establishing these thresholds will enable trait-based strategies to incorporate predictive, mechanistic criteria, supporting species-specific timelines for secure attachment and guiding when, where, and how to plant different taxa [77].

## Data Availability

The data supporting the findings of this study are openly available in the Dryad Digital Repository [78]. Supplementary material is available online [79].
